# Research on Total Internal Reflection Detection Technology for Subsurface Defects of Optical Elements Based on Spectral Confocal Principles

**DOI:** 10.3390/s25133969

**Published:** 2025-06-26

**Authors:** Rongcai Bao, Kaige Qu, Lu Wu, Shijian Zhang, Anyu Sun

**Affiliations:** College of Engineers, Robotics and Intelligent Manufacturing Engineering, Zhejiang University, Hangzhou 310000, China; 22460237@zju.edu.cn (R.B.); 12025074@zju.edu.cn (L.W.); 22260022@zju.edu.cn (S.Z.); anyusun@zju.edu.cn (A.S.)

**Keywords:** subsurface defect, nondestructive testing, spectral confocal, total internal reflection

## Abstract

During the manufacturing of precision optical elements, subsurface defects seriously affect the performance of the elements, leading to the enhancement of light fields, an increase in laser absorption and an decrease in mechanical properties. It has become a key technology to realize the high-precision quantitative automatic detection of subsurface defects of optical elements. This paper presents a method of subsurface defect detection based on spectral confocal scattering measurement, the system adopts a dispersive lens group with the working band of 480–670 nm, and combines the spectral confocal technology and total internal reflection technology to effectively suppress the interference of scattered light on the surface, and can realize high-precision nondestructive detection without fluorescent substances. The axial resolution of this method is 0.8 μm and the measuring depth range is 0.94 mm. By building a measurement system and carrying out experimental verification, the results show that this method can accurately measure the depth and location of subsurface defects and confirm its feasibility and effectiveness.

## 1. Introduction

As the key process of surface machining, grinding and polishing of optical elements are the main sources of subsurface defects [1,2,3,4,5,6,7]. Movement of abrasive particles in grinding produces microcracks on the surface of materials, and these initial cracks may extend to deeper subsurface areas under shear stress, eventually forming a penetrating deep crack network [8], thus affecting the microstructure stability and service life of materials [3]. Non-destructive testing technology has developed rapidly because of its characteristics of protecting the surface integrity of specimens, convenient operation and non-contact measurement. During the evaluation of surface and subsurface damage [9], these techniques can detect tiny inhomogeneities in materials, deduce the relationship between them and physical parameters, and then realize high-precision evaluation of damage depth, crack distribution and characteristics.

Optical coherence tomography (OCT) [10] is based on the principle of low coherence interference, and has micron resolution. Time-domain OCT can reconstruct cross-sectional images, and frequency-domain OCT can extract depth information through Fourier transform, which can improve imaging speed, signal-to-noise ratio and spatial resolution without mechanical scanning. However, when OCT detects optical materials, the subsurface damage signal is very weak, and it is difficult for traditional time-domain or frequency-domain OCT to generate enough interference signals, which significantly limits its detection effect.

In quantitative assessment, Li’s team [5] developed a theoretical model correlating subsurface damage with surface roughness by analyzing median and lateral crack systems induced by sharp indenters on brittle surfaces. They validated the model’s effectiveness using magnetorheological finishing and contact profilometry to measure subsurface damage depth and surface roughness in BK7 glass before and after grinding. However, this predictive model has limitations: it lacks a theoretical basis for equating lateral crack length with surface roughness P-V values, and its simplification of grinding/polishing processes into indenter pressing models significantly deviates from actual manufacturing conditions, restricting the model’s reliability and applicability.

Total internal reflection microscope (TIRM) [11] is based on total internal reflection effect, which can identify surface/subsurface micro-defects of materials and has outstanding advantages in resolution and detection sensitivity. Cui Hui [12] combined with digital image technology to expand the application of TIRM, which can analyze the internal defect characteristics of optical elements and accurately locate the spatial position and size of defects. However, TIRM has inherent disadvantages: diffraction effect reduces the lateral resolution, resulting in the measured value of defect size being larger than the actual value. The longitudinal resolution is limited by the accuracy of positioning platform, the resolution of microscope and the depth of focus.

Confocal laser scanning microscopy (CLSM) [13,14,15] adopts a pinhole conjugate optical path design, where only the scattered light from the focal plane can reach the detector, and three-dimensional imaging of the sample [16] can be realized through XY plane scanning and Z axis movement. This technology is mature in the field of bioluminescence imaging, but it faces a key bottleneck when it is used to detect subsurface defects of optical materials such as molten Shi Ying: the scattering signal of micron defects in transparent media is weak, resulting in an insufficient signal-to-noise ratio [17]. The existing fluorescence labeling method [18] enhances the signal, but the sample preparation is complicated and the distribution uniformity of fluorescent materials seriously affects the effect. In addition, the resolution of the CLSM system is limited by the numerical aperture of the objective lens, and the residual fluorescent materials introduce additional quantitative errors.

To overcome the limitations of existing technologies, this paper proposes a novel subsurface defect detection method combining spectral confocal and total internal reflection (TIR) techniques. The method achieves high-precision detection with an axial resolution of 0.8 μm. It operates over a depth range of 0.94 mm and requires no fluorescent agents. The key improvements include the following:

(1) The integration of TIR technology effectively addresses the limitations of conventional confocal techniques in non-fluorescent sample detection.

(2) The TIR mechanism significantly suppresses surface-scattered light, ensuring that the detected signals primarily originate from internal defect scattering. This enhancement substantially improves signal purity and signal-to-noise ratio.

(3) Compared with traditional TIR microscopy (TIRM), our approach incorporates spectral confocal technology through a dispersive lens system. This innovation focuses different wavelengths at distinct spatial positions, enabling wavelength-specific detection through the pinhole aperture. The combined system achieves superior resolution and sensitivity in defect detection.

## 2. Principle and System Design

### 2.1. Scattering Analysis of Total Internal Reflection Defects

**Figure 1:**
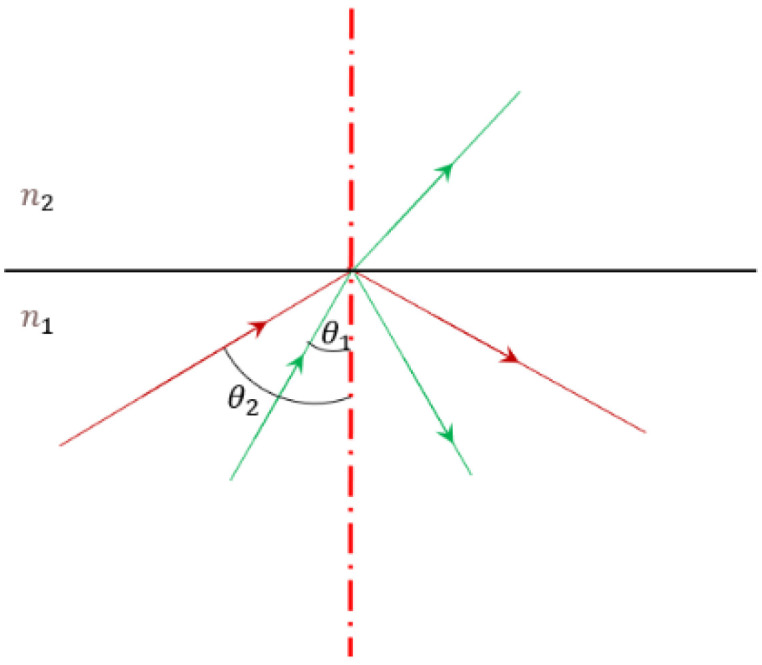
Schematic diagram of total internal reflection.

Figure 1 shows that when light travels from a medium with a higher refractive index (an optically dense medium) into a medium with a lower refractive index (an optically less dense medium), a critical angle θ for total reflection exists. If the incident angle is less than this critical angle, the light will be both reflected and refracted. When the incident angle equals the critical angle, the refraction angle α reaches 90 degrees, the critical state. If the incident angle increases further, the incident light will be totally reflected.

When parallel light enters the sample and focuses on a defect point, it will excite scattering light. The intensity of the scattering light is usually a small fraction of the incident luminous intensity, with the specific proportion depending on several factors, including the size, shape and material properties of the defect, the wavelength of the light and the scattering angle. When the wavelength is much larger than the defect size, it is typically Rayleigh scattering; when the wavelength is comparable to the defect size, it is Mie scattering; and when the wavelength is much smaller than the defect size, it is geometric optics scattering.

Rayleigh scattering occurs when the wave length is larger than the defect size:

(1) $$\frac{I\prime }{I}=\left(\frac{1+\cos^{2}\theta }{2}\right)\left(\frac{1}{\lambda^{4}}\right)$$

where I is the incident luminous intensity, θ is the scattering angle, λ is the wavelength, and $\frac{I\prime }{I}$ is usually in the range of $10^{-6}$ to $10^{-2}$.

Mie scattering occurs when the wavelength is equal to or close to the defect size:

(2) $$\frac{I\prime }{I}\alpha Q_{sca}$$

where $Q_{sca}$ is the scattering efficiency, which usually ranges from $10^{-2}$ to $10^{-1}$.

It can be observed from Formula (1) that both Rayleigh scattering and Michaelis scattering show significant directional differences: the efficiency of forward scattering (the direction of scattered light is the same as that of incident light) is usually twice that of backward scattering (the direction of scattered light is opposite to that of incident light). In Michaelis scattering, when the size of defect particles is significantly larger than the wavelength of incident light, most of the light energy is concentrated in the forward direction. When the particle size is equivalent to the wavelength, the forward scattering intensity is usually more than ten times that of the backward scattering. This characteristic leads to that when the light focuses on the defect and scatters, and the intensity of the backscattered light itself has been attenuated by more than ten times. A confocal scanning optical system faces essential challenges because it needs to detect the backscattering part of scattered light: backscattering only accounts for a small part of the total scattered light, and is limited by the size of the detection pinhole and the imperfection of the optical imaging system, and the reflected light on the sample surface will be received by the sensor. These factors together make it difficult for the system to effectively capture weak scattering signals from internal defects.

Therefore, this technology often needs to be combined with fluorescence imaging; by adding a fluorescent agent to the grinding liquid, it depends on the fluorescence signal excited by the defect in how it is measured. However, this method is not really nondestructive testing: it is necessary to introduce exogenous fluorescent substances, and it is also impossible to detect defects without fluorescent substances. The key scheme to solve the above problems is to introduce the mechanism of total internal reflection (TIR). When the incident angle is greater than the critical angle, the light is totally reflected at the interface and does not enter the low refractive index medium. If there is no TIR condition, the interaction between the light and sample surface will produce a lot of surface scattered light, which will seriously interfere with the detection of scattered light from internal defects. The introduction of TIR effectively inhibits the surface scattering/reflection: the incident light meeting the TIR condition is completely reflected back to the original medium and cannot enter the focusing optical path, and only the scattered light generated by internal defects may penetrate the interface and be selectively captured by the optical system.

### 2.2. Principle of Chromatic-Defocusing and Calibration Basis

**Figure 2:**
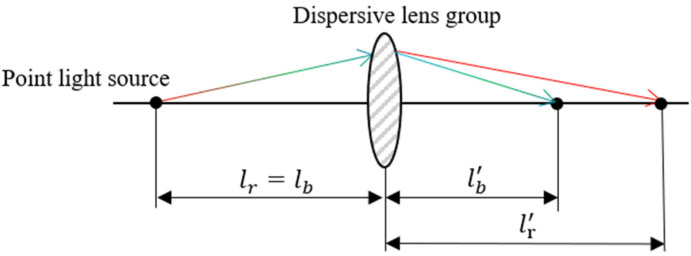
Schematic of chromatic focal shift.

Figure 2 shows the chromatic focal shift principle: a point source emits visible light that serves as the object for the optical system. Since different wavelengths exhibit varying refractive indices in the dispersive lens assembly, the polychromatic beam forms multiple focal points after passing through the chromatic objective [19,20]. In Figure 2, $l_{r}$ and $l_{b}$ represent the object distances for red and blue light, respectively, while $l_{r}^{\prime }$ and $l_{b}^{\prime }$ denote their corresponding image distances.

Based on the principle of dispersion shift, spectral confocal ranging technology uses a wide-spectrum white light source to axially separate different wavelengths of light through a dispersion system to form a continuous focal array. When the measured object is located in the working area, the light with a specific wavelength is focused on its surface, and the reflected light returns through the original path. The core of the system lies in the conjugate relationship between the pinhole and the dispersive objective lens: only the surface-focused wavelength light can be detected by the spectrometer through the pinhole, and the other wavelengths are shielded. Through the pre-calibrated wavelength–depth mapping relationship, the detection wavelength can be converted into accurate position information.

### 2.3. Subsurface Defect Depth Model

**Figure 3:**
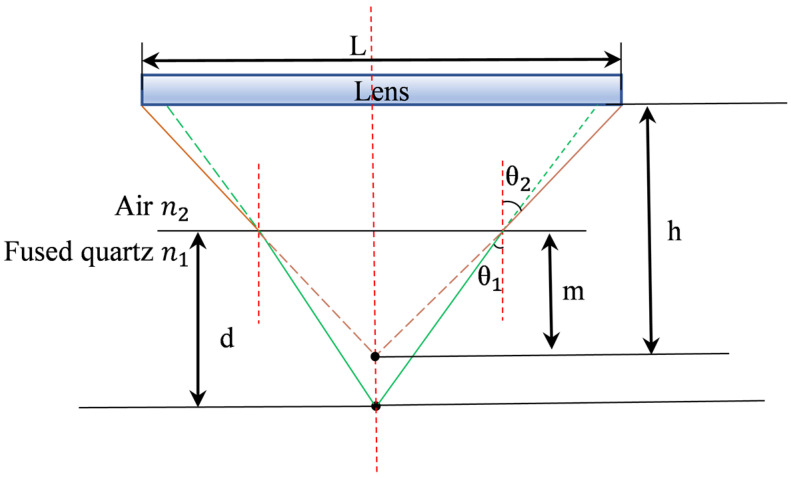
Internal depth scanning measurement model.

As shown in Figure 3, scattered light is excited by a defect point located in the interior of the sample with a distance d from the upper surface, and the light is refracted on the upper surface of the sample, so that the depth calculated by the detection system through direct fitting is m. It is necessary to convert m into d according to the depth model, and take air refractive index $n_{2}$, Shi Ying glass refractive index $n_{1}$, scattered light incident angle $\theta_{1}$ and refraction angle $\theta_{2}$ as follows:

(3) $$\left\{\begin{array}{c} n_{1}\sin\theta_{1}=n_{2}\sin\theta_{2}=NA\\ m\tan\theta_{2}=d\tan\theta_{1} \end{array}\right.$$

where

(4) $$d=m\times \left(\sqrt{\frac{n_{1}^{2}-NA^{2}}{n_{2}^{2}-NA^{2}}}\right)=m\times K$$

Through this model, we can know the proportional relationship between the true depth d of defects and the direct fitting result m, in which the coefficient K is not a constant, which is related to the receiving wavelength and the sample properties.

**Figure 4:**
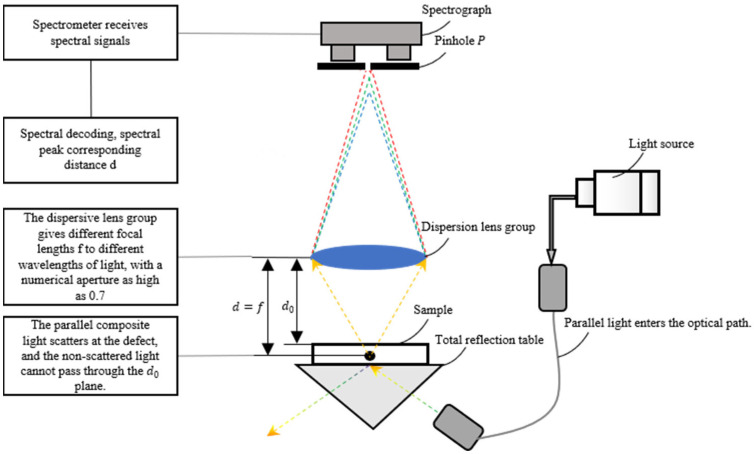
Schematic diagram of subsurface defect detection of optical elements.

Based on the above principles, this paper proposes a method for detecting subsurface defects of optical components based on spectral confocal and total internal reflection (TIR). As shown in Figure 4, after combining the parallel excitation light (essentially high-intensity forward scattered light) with the total internal reflection optical path design, the focused optical path can effectively eliminate the interference of surface scattered light, so that the signals received by the spectrometer are completely scattered by internal defects. During detection, a beam of parallel light enters the sample. When the parallel light reaches the upper surface $d_{0}$ of the sample, because its incident angle is greater than the total reflection angle, the parallel light will be totally reflected and will not enter the focusing system through the surface. However, when there are defects (such as cracks) in the sample, the defects will scatter light, and part of the scattered light can pass through the surface $d_{0}$. Because the focusing system uses a dispersive mirror, only the light with a specific wavelength can be completely focused on the pinhole P, so the spectral signal received by the spectrometer can decode the distance d, thus realizing the measurement of subsurface defects.

### 2.4. System Design

**Figure 5:**
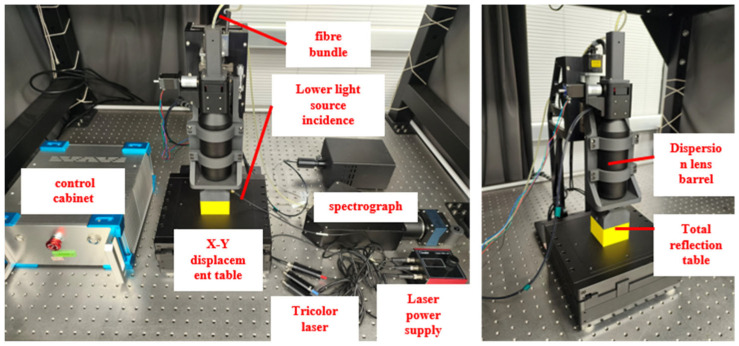
Physical diagram of total internal reflection spectrum confocal detection system.

Figure 5 shows the setup of a spectral confocal scattering measurement system. This system refers to the microscopic technology of confocal measurement of subsurface defects, introduces the spectral confocal technology to obtain the axial position information of subsurface defects and then introduces the total internal reflection technology to eliminate the interference of incident light source, this leads to the development of spectral confocal scattering measurement technology that can directly and nondestructively detect subsurface defects in the sample under test without the need for fluorescent substances, and theoretically designs the detection system. Based on the design theory and actual situation, this section completes the overall design of the detection system, including the detailed design of light source, dispersive confocal lens group [21,22,23], total internal reflection system and spectral signal receiving and imaging system, and completes the model construction and processing and assembly of the whole system.

Before the experiment, it is necessary to calculate the critical angle of the sample–air interface to provide a theoretical basis for optimizing the selection of incident angle, and determine the axial resolution of the system to represent the measurement accuracy limit of defect depth detection in the experimental system.

(1) Determine the incident angle [1]:

(5) $$\sin(\theta_{c})=\frac{n_{2}}{n_{1}}$$

where $n_{1}$ is the refractive index of the sample (fused quartz) and $n_{2}$ is the refractive index of the prism, the calculated critical angle $\theta_{c}$ is about 43.3, 45 is selected as the incident angle.

**Figure 6:**
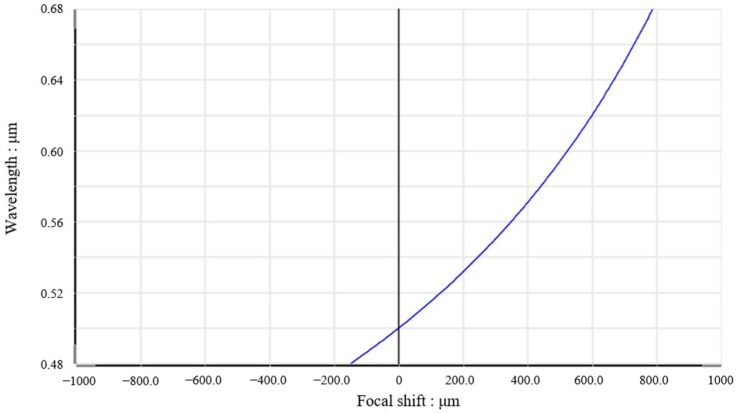
Chromatic focal shift diagram.

(2) Axial resolution of measuring system:

As shown in Figure 6, the working wavelength range of the dispersion mirror set designed in this paper is 480–670 nm, the axial measurement range is 0.94 mm and the high numerical aperture (NA) is 0.7. The transverse resolution of the dispersive lens group only depends on the focusing wavelength λ, and the smaller the wavelength, the higher the resolution, ranging from 0.2 µm to 0.4 µm (the transverse resolution is 0.28 µm at 500 nm). Its axial resolution decreases with the increase in wavelength, ranging from 0.1 µm to 0.2 m (axial resolution is 0.13 µm at 500 nm wavelength). However, the ultimate axial resolution of the whole detection system mainly depends on the performance of the spectrometer. The spectrometer used in this experiment has an axial displacement of about 1 mm at the wavelength of 480 nm to 720 nm, and the relationship between wavelength and axial displacement is approximately linear, resulting in an axial resolution of 0.2 nm from the wavelength resolution of the spectrometer. Therefore, the axial resolution of this experimental system is 0.8 µm.

**Figure 7:**
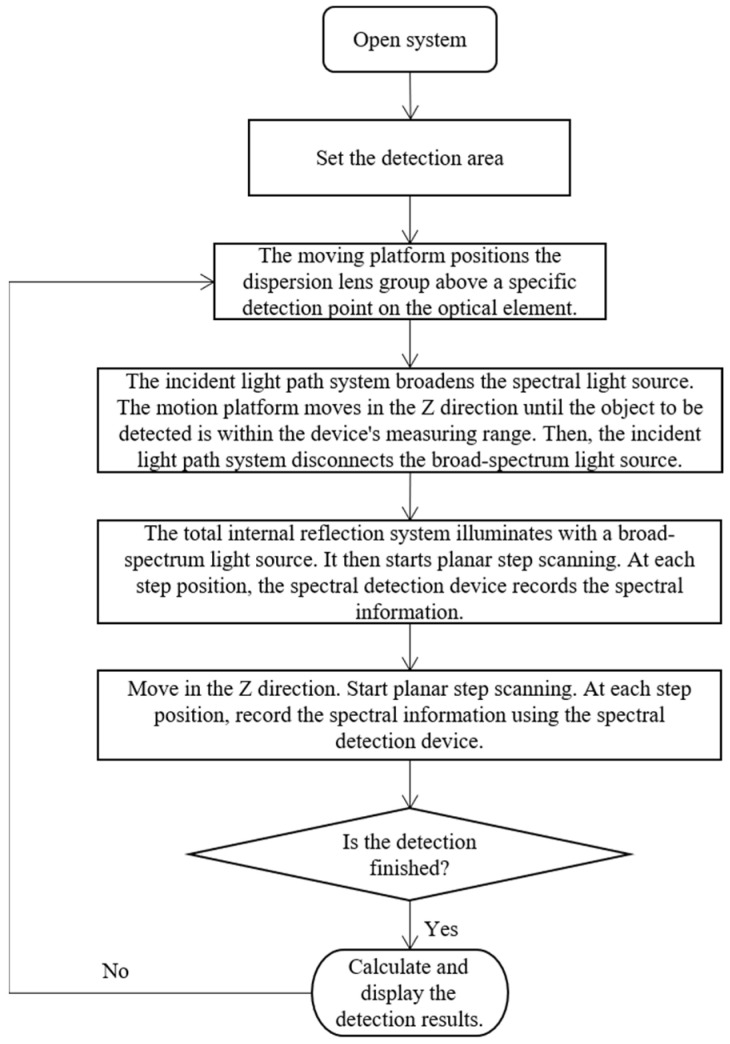
Flow chart of subsurface defect detection.

When detecting subsurface defects with the detection system, the whole process can be divided into two main steps: hardware scanning and software processing. As shown in Figure 7, the overall process of hardware scanning needs to determine the scanning parameters, including scanning area, stepping interval and camera parameters. After scanning the subsurface region of the sample is completed, the software processing stage is entered. At this stage, it includes image processing, spectrum extraction, coordinate calculation and three-dimensional shape fitting output.

## 3. Experimental Verification

### 3.1. Data Processing

Data processing is the key step to achieve high precision measurement in spectral confocal scattering measurement. Here are the specific steps of data processing: when the total internal reflection system broadens the spectral light source, record the spectral information as Q:

(6) $$Q=\{q(x_{0},y_{0}),q(x_{0},y_{1})\ldots q(x_{n},y_{n})\}$$

$\left(x_{n},y_{n}\right)$ is the plane coordinate of the nth scanning point. Based on the principle of total internal reflection, the incident polychromatic light cannot pass through the upper surface of the object to be measured. However, the subsurface defect area will scatter the light, and some of the scattered light can penetrate the upper surface of the object. Based on the basic characteristics of the dispersive focusing optical path, the slit mainly receives the scattered light with a specific wavelength corresponding to the object distance of the defect point and guides it to the spectrum detection device. Then, the collected scattered light contains spectral information $q(x_{n},y_{n})$:

(7) $$q(x_{n},y_{n})=\int_{\lambda 0}^{\lambda_{k}}I(\lambda )d\lambda$$

In Formula (7), λ is the wavelength of light, I(λ) is the luminous intensity value of the wavelength λ. In the image output by the spectral detection device, q corresponds to a horizontal bright line, the horizontal coordinate a of the bright line corresponds to the wavelength λ, and the gray value G(a) of the bright line corresponds to $E_{\lambda }$, therefore

(8) $$q(x_{n},y_{n})=\int_{a_{0}}^{a_{k}}G(a)da$$

The spectral detection device generates an image. It samples the gray value of this image to obtain the corresponding spectral information. The working target surface of the device contains k pixels in the transverse direction, allowing for the extraction of $q(x_{n},y_{n})$.

Under the dispersion shift principle, the detection optical path mainly captures the scattered light from the conjugate point corresponding to a specific wavelength. The wavelength affects the position of the conjugate point, causing light of different wavelengths to form corresponding scattering responses in the optical path. The function image of $q(x_{n},y_{n})$ is a single-peak spectral curve, and the transverse coordinate of the peak $a^{\prime }$ corresponds to the object distance p of the defect point.

(9) $$p=f(a^{\prime })$$

In Formula (9), $f(a^{\prime })$ is the calibration function that relates the transverse coordinates of the peak to the object distance.

Following these steps, we can calculate the defect position for each scanning point. Finally, integrating the defect position information from all scanning points yields the three-dimensional shape of subsurface defects.

### 3.2. Calibration Experiment

**Figure 8:**
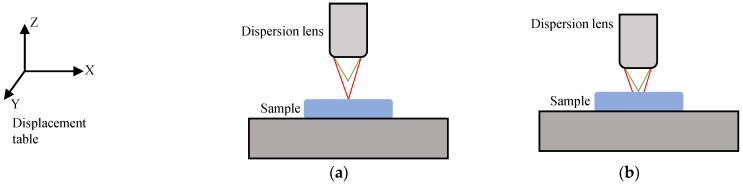
Spectral calibration experiment of detection system. (**a**) the Z-direction position of the lens group when the red light is focused. (**b**) Z-direction position of the lens group when the green light is focused.

In order to make the broad spectral information received by the spectral detection device correspond to the coordinate information of the reflecting surface, it is necessary to decode the spectral data. Accurate spectral decoding needs to rely on calibration experiments, remove the precision of peak wavelength extraction and obtain the accurate distance from the reflecting surface to the lens. The specific steps for measuring the calibration data are as follows: As shown in Figure 8, the Z axis of the displacement platform drives the dispersive lens to move 10 µm at a time, and the measurement is repeated for 10 times for each calibration point, and the spectral data and displacement value of each time are recorded with a calibration distance of 1.5 mm (limited by the dispersion focal shift range of the dispersive lens group under the white light source). After obtaining the calibration data, the system tests and demonstrates the calibration results.

**Figure 9:**
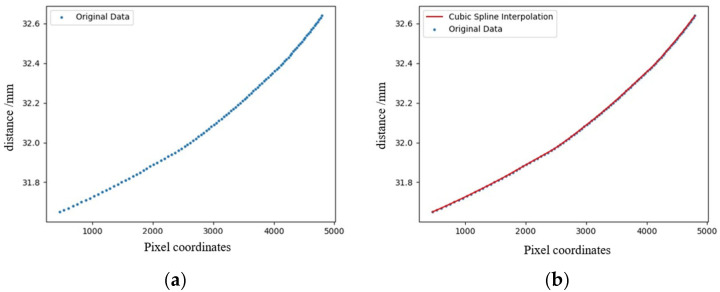
Results of calibration experiment: (**a**) coordinate distribution map corresponding to peak wavelength, and (**b**) cubic spline interpolation fitting map of wavelength coordinates.

The CMOS camera used has a transverse pixel count of 6460, meaning the spectral image contains 6460 points. After removing dark signals and applying wavelet denoising, the system performs Gaussian fitting to obtain the peak wavelength corresponding to each displacement coordinate (when white light enters the detection system and is dispersed by a blazed grating, different wavelengths of light are distributed laterally and reach the CMOS, so each transverse pixel in the captured image receives light of a fixed wavelength). Figure 8a shows a scatter plot of different peak wavelength positions and distances. Figure 9a shows the calibrated original recorded data. Figure 9b shows the fitting results after applying cubic spline interpolation fitting to the wavelength coordinate points and saving the fitting results, completing the calibration.

**Figure 10:**
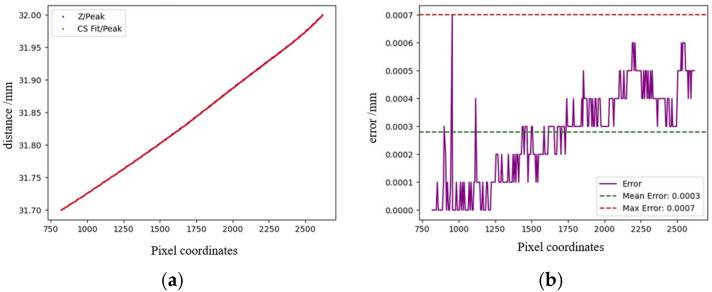
Fitting error analysis: (**a**) blue is the actual value of Z coordinate, and red is the fitting value. (**b**) The data in the figure are the errors between the actual value and the fitting value.

Next, prove the calibration results, arbitrarily choose 300 µm in the working range of the detection system, collect the reflection images step by step and obtain the fitting coordinates through data processing. Figure 10 shows the calibration results; after a multi-stage demonstration, the error between the fitting results obtained through cubic spline interpolation and the actual values is less than 1 m, which satisfies the experimental requirements.

### 3.3. Sample Preparation and Treatment

Grinding and polishing is a key process in optical manufacturing and the primary source of subsurface defects in optical processing. Its main purpose is to remove surface scratches left from the cutting process, further refine the workpiece’s geometric dimensions, improve flatness and reduce surface roughness, thereby providing a better foundation for subsequent polishing [24,25,26]. This study used a UNIPOL-1200S automatic grinder and polisher for the experiments. The experiments involved grinding and polishing circular glass wafers. The grinding parameters were set as follows: the turntable rotation speed was set to 80 r/min, the grinding wheel rotation speed was set to 50 r/min, and the grinding pressure was set to 0.5 kg. The grinding particles used were silicon carbide with a particle size of 300 µm, corresponding to 800 mesh, and the grinding time was set to 20 min.

**Figure 11:**
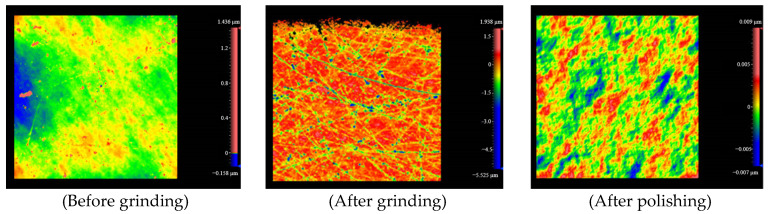
White light interferometry chart of sample surface.

To confirm that the sample surface is free of defects and to prevent the detection device in this study from mistakenly identifying surface defects as subsurface defects, a white light interferometric microscope was used to measure the sample surface. Figure 11 shows the surface morphology of the sample after grinding and polishing, and the final optical element sample obtained has no surface defects larger than 1 µm.

### 3.4. Experiment of Subsurface Defect Detection

The detection system is used to inspect subsurface defects in the ground samples. Since the locations of subsurface defects are unknown, the surface should first be scanned to determine its topographical features and eliminate interference from surface defects. Subsequently, a layer-by-layer coarse scan is performed. By examining the coarse scan images of each layer, potential subsurface defect locations can be identified. Fine scans are then conducted at these suspected subsurface defect locations.

**Figure 12:**
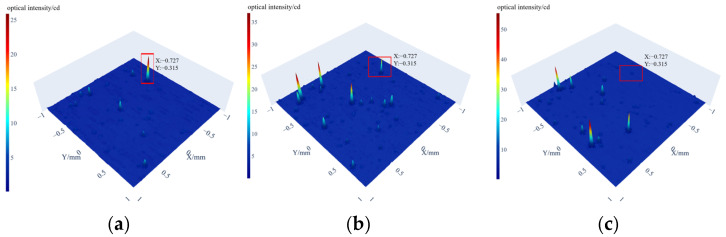
Layer-by-layer scanning diagram of scattered light distribution: (**a**) depth 20 µm, (**b**) depth 40 µm and (**c**) depth 60 µm.

Next, fix the sample to be tested on the total reflection table, fill the gap between the bottom of the sample and the triangular prism of the total reflection table with castor oil, and plan a square scanning area with a central side length of 2 mm. Surface characterization shows all surface defects measure below 0.1 μm after sample preparation. The next step is to detect subsurface defects. Turn on the laser light source connected to the lower light source in Figure 4. The incident laser enters the total reflection table, and the system scans the surface of the ground and polished sample layer by layer to observe the scattered light distribution of each layer. In Figure 12, three pictures show the scattered light distribution at three different depths, and (*x*, *y*) in each picture is the plane coordinate, and the *z* value is the received scattered luminous intensity value; positions with higher luminous intensity values may indicate the presence of defect characteristics in the three pictures. Select a defect feature for precise scanning.

**Figure 13:**
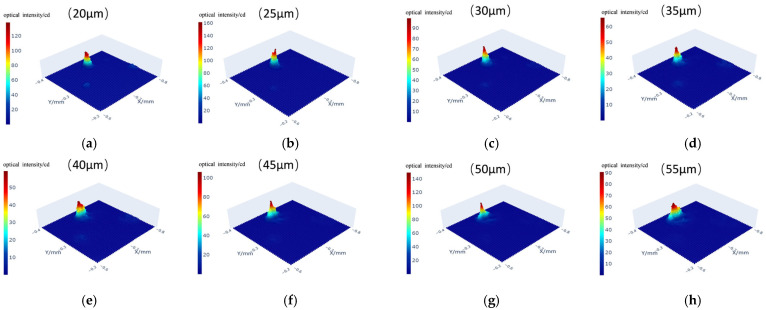
Fine layer-by-layer scanning diagram of subsurface defects. (**a**) 20 µm; (**b**) 25 µm; (**c**) 30 µm; (**d**) 35 µm; (**e**) 40 µm; (**f**) 45 µm; (**g**) 50 µm; (**h**) 55 µm.

Select regions of X: −0.6 to −0.8 mm and Y: −0.2 to −0.4 mm from the coarse scanning image for fine scanning of subsurface defects. Set the step interval in the X-Y plane to 5 µm and start scanning from a depth of 20 µm, with each layer separated by 5 µm. Figure 13a–h show the intensity maps for the first 8 layers, from 20 to 55 µm depth.

Regard the subsurface defect as a collection of several spatially discrete defect points, regard the axial distance between the position and the scanning depth of a defect point as d, and d is a positive value, then the scanning luminous intensity value of the plane position corresponding to the defect point increases with the decrease in d. Based on this conclusion, the laser scanning data are processed as follows:

I. The original data obtained by the scanning system are grouped according to the plane coordinates (x,y), and the depth–optical intensity sequence (z,E) of each position point is obtained, where z is the axial position recorded by the scanning control system (which can be converted into the actual depth) and e is the scattered optical intensity value detected at the corresponding depth z.

II. The peak value of the depth–intensity sequence at each (x,y) position is analyzed, and the judgment conditions are as follows: there is an obvious maximum intensity $E_{p}$, and the intensity on the left side shows an upward trend with the increase in depth, and on the right side shows a downward trend with the increase in depth. The (x,y) is the coordinate of the defect point when the above conditions are met.

**Figure 14:**
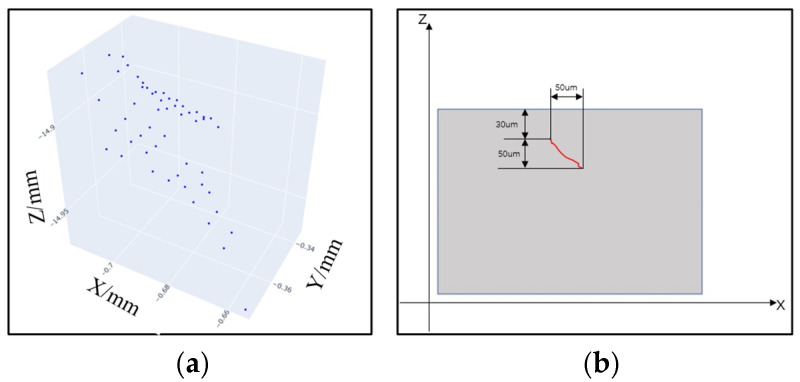
Scanning results of subsurface defects: (**a**) point clouds of subsurface defect points, and (**b**) schematic diagram of the position characteristics of the defect.

According to the calibration curve established by the calibration experiment, the peak optical intensity $E_{p}$ of the defect point is converted into the actual depth of the defect, processing all points in the plane. Figure 14a shows the point cloud image of the subsurface defect, and fitting the three-dimensional shape. Figure 14b shows the position feature of the defect, and obviously, the defect is a typical crack feature.

**Figure 15:**
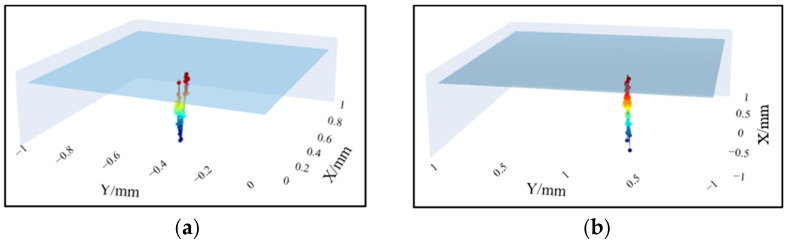
Detection results of subsurface defects at different positions. (**a**) Subsurface defect at position A. (**b**) Subsurface defect at position B.

Select the remaining suspected subsurface defects in Figure 10 for fine scanning, and Figure 15 shows the subsurface cracks with different positions and shapes.

## 4. Conclusions

This paper presents a method for detecting subsurface defects of optical components based on spectral confocal and total internal reflection technology. This method can realize high-precision nondestructive testing without fluorescent substances. The detection system adopts a dispersive lens with the working wavelength range of 480–670 nm, the axial range of 0.94 mm and the numerical aperture (NA) of 0.7, and the axial resolution can reach 0.8 micron. The experimental results show that the system can accurately measure the depth and location of subsurface defects. The future work will focus on optimizing the system design, verifying its applicability to a variety of optical materials and clarifying the improvement range of the signal-to-noise ratio compared with traditional detection methods, so as to further improve the detection accuracy and adaptability.

## Data Availability

The data presented in this study are available on request from the corresponding author.
